# A CDC42 Stop-loss Mutation in a Patient with Relapsing Polychondritis and Autoinflammation

**DOI:** 10.1007/s10875-022-01344-z

**Published:** 2022-08-30

**Authors:** Rogier T. A. van Wijck, Sigrid M. A. Swagemakers, Peter J. van der Spek, P. Martin van Hagen, Paul L. A. van Daele

**Affiliations:** 1 Department of Pathology & Clinical Bioinformatics, Erasmus University Medical Center, Rotterdam, The Netherlands; 2 Department of Internal Medicine, Division of Allergy & Clinical Immunology, Erasmus University Medical Center, Rotterdam, The Netherlands; 3 Department of Immunology, Erasmus University Medical Center, Rotterdam, The Netherlands

To the Editor,

Cell division control protein 42 homolog (CDC42) is one of the most well-studied members of the Rho GTPase family. Rho GTPases have a critical role in a wide variety of pivotal cellular functions like rearrangement of the actin cytoskeleton, cell polarity, cell motility, vesicle trafficking, cell cycle regulation, transcription activation, and migration [1]. Mutations in *CDC42* are associated with a clinically heterogeneous group of phenotypes, including growth retardation, neurodevelopmental anomalies, cardiac malformations, immune dysregulation, and hematological disturbances (Table S1) [2]. Martinelli and colleagues classified the *CDC42* variants in three groups based on their position in the *CDC42* structure and their functional characterization. Recently, variants in the C-terminal region of *CDC42* have been described, which make up a fourth group of mutations. These mutations cause an autoinflammatory syndrome characterized by neonatal onset of fever, rashes, and cytopenias ultimately leading to secondary hemophagocytic lymphohistiocytosis (HLH) in a significant number of patients.

We report the oldest patient with a known pathogenic mutation in *CDC42* diagnosed with relapsing polychondritis (RP) and autoinflammation, who succumbed at the age of 55 from multiorgan failure. At age 42, she presented to the clinical immunological department with short stature resembling achondroplasia (height of 134 cm), subtle dysmorphic features (round face with flat profile, mild upslanting of her palpebral fissures, and a wide nasal tip), hepatosplenomegaly, subglottic stenosis, scleritis, and inflammation of both auricles. From childhood onwards, she suffered from recurrent infections including multiple pneumonias and an episode of viral meningitis. She worked as an artist and had a clinical history of asthma and progressive bilateral sensorineural deafness since early adulthood. Neurologically, she was completely normal and brain MRI showed no structural abnormalities. Immunoglobulin screening at the time of presentation showed an isolated IgM deficiency (< 0.3 g/L; ref 0.45–2.3 g/L) with normal IgG and elevated IgA (3.9–22.0 g/L; ref 0.76–3.91 g/L). Other laboratory evaluations revealed an increased ESR and CRP, mild anemia, and slightly elevated liver enzymes. Auto-immune serological tests were negative. No thrombocytopenia or leukopenia were observed; however, there was a slight decrease of B-cells (0.07 × 10^9^; ref 0.1–0.4 × 10^9^) and NK-cells (0.02 × 10^9^; ref 0.1–0.4 × 10^9^). Bone marrow biopsy revealed hypercellularity with trilineage hematopoiesis interpreted as reactive changes. Over time, she was admitted multiple times for pneumonias and treated with antibiotics. She was diagnosed as RP and suspected from an immunodeficiency. She was treated with glucocorticosteroids and IgG maintenance therapy. When she was 44, a partial jejunum resection was performed after she suffered from acute abdominal pain due to intestinal necrotizing vasculitis. At age 55, she was admitted with acute abdominal pain from a suspected ileus. During admission, she worsened and died due to hemodynamic instability and pulmonary hypertension; additionally, during autopsy, a persistent foramen ovale was found.

Because of the complex nature and severity of the disease, WES was performed on whole blood DNA of the patient after informed consent was obtained. Analysis revealed a heterozygous stop-loss mutation (NM_001039802, c.576A > C; p.*192Cys*24, gnomAD frequency, never observed) in *CDC42* (Fig. 1b), which causes an abnormal addition of 24 amino acids to the *CDC42* protein. The specific mutation we found was previously described in a patient with neonatal-onset multisystem inflammatory disease (NOMID), an IL-1-mediated autoinflammatory disease [3]. Recently, functional analysis of this particular mutation showed impaired geranyl-geranylation resulting in Golgi-restricted mislocalization of the mutant protein, hyperactivation of the pyrin inflammasome, and subsequent accelerated proteasomal degradation [4, 5]. Moreover, we also found extremely rare and severe truncating heterozygous mutations in *PANX3* (NM_052959 c.765delC; p.V255fs, gnomAD frequency 3.98 × 10^–6^) and *JDP2* (NM_001135049 c.476dupC; p.T159fs, gnomAD frequency 4.00 × 10^–6^), which both play a role in cartilage and bone development. Unfortunately, her parents were already deceased at the time of these findings, so we could not determine the de novo status of these mutations. However, we performed Sanger sequencing in her immunologically healthy sister for the observed variants and all were absent. Therefore, we could not rule out a contributing and modifying role for these rare variants.

**Fig. 1:**
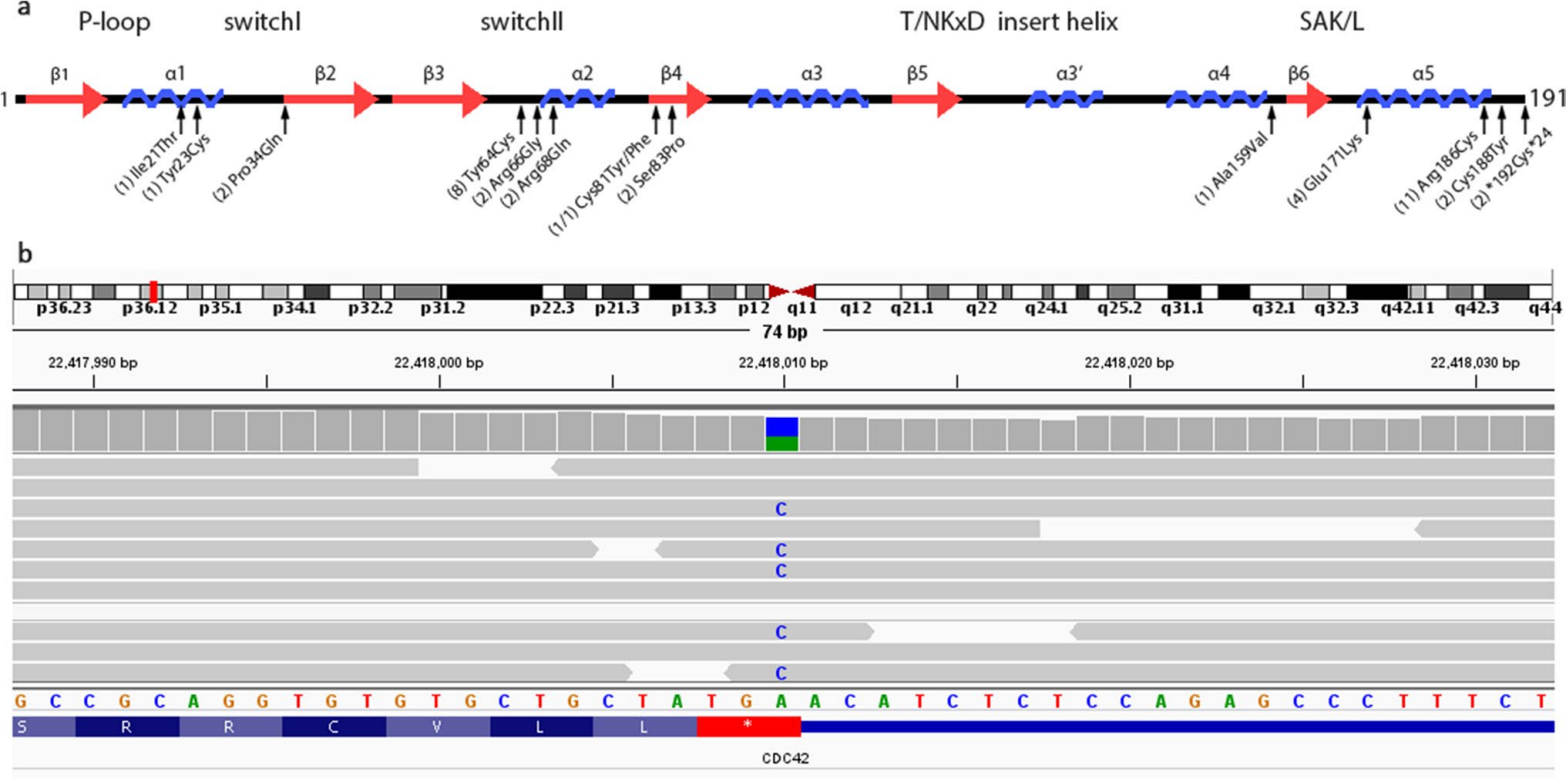
**a** Schematic representation of the secondary structure (α-helices and β-strands) of *CDC42* and the positions of known disease-causing *CDC42* mutations and their reported frequency. The variant reported here (c.576A > C; p.*192Cys*24) is included in this figure. **b** The heterozygous c.576A > C mutation at the final codon of *CDC42* results in read-through of the stop codon and adds 24 amino acids to the C-terminus of the CDC42 protein

RP is a rare progressive inflammatory disorder that affects the cartilaginous tissue throughout the body. It is characterized as an episodic immune-mediated condition most often affecting the ears, nose, joints, and respiratory tract, but it can also have systemic and ocular manifestations. The exact etiology and pathogenesis remain elusive, partly because of the rareness and heterogeneity of the condition. However, there is evidence for a cell-mediated autoimmune reaction and autoantibodies matrix proteins like type II, IX, and XI collagen or matrilin-1 and cartilage oligomeric matrix proteins (COMP). Recently, somatic mutations in the X-linked gene *UBA1* have been associated with chondritis. Moreover, RP is associated with several immunologic and hematologic conditions, suggesting an underlying immunologic mechanism.

Currently, at least 39 patients have been described in literature with mutations in *CDC42* (Fig. 1 and Table S1). A broad spectrum of clinical phenotypes is associated with these variants. Patients can be roughly categorized into four groups based on the pathogenic mutation. Most pathogenic *CDC42* mutations have been investigated functionally, showing a disrupted protein function of CDC42, including impaired binding between CDC42 and its regulators and effectors, hyperactivity of nucleotide exchange resulting in increased CDC42 signaling, and aberrant palmitoylation and geranyl-geranylation leading to mislocalization of CDC42 (reviewed in [1]). These specific consequences likely do account for the variable clinical phenotypes associated with *CDC42* variants. Our patient harbored the p.*192Cys*24 mutation in the C-terminal of *CDC42*. She presented with systemic autoinflammatory disease, a characteristic finding of C-terminal mutations. Similar to other patients, she also presented with short stature, recurrent infections, hepatosplenomegaly, and transaminitis. However, unlike other patients with C-terminal mutations, her disease was not neonatal onset nor did we observe severe cytopenias. Differences in phenotype may be explained by the level of detailed phenotypic description of patients, the occurrence of rare variants in additional genes that can modify the clinical phenotype and the age of disease presentation. However, despite an intricate genotype–phenotype relationship, some intragroup variability is also observed as highlighted by this and other cases [2].

*CDC42* is critical for cytoskeletal rearrangement, which affects cell mobility and migration. Cell motility is driven by cytokines and chemokines and is essential for the immune system to adequately respond to pathogens. Moreover, an impaired actin cytoskeleton also reduces the ability of immune cells to adequately invaginate the plasma membrane necessary for endocytosis, phagocytosis, and formation of the immunological synapse. Mutations and defects in genes that play a role in actin cytoskeleton rearrangement have been described in primary immunodeficiencies such as *WASP* and *DOCK8*. Complete aberration of *CDC42* is lethal suggesting a crucial role of *CDC42* in normal development. Unfortunately, mammalian models carrying heterozygous *CDC42* mutations are still lacking; however, knock-out models of Cdc42 have been studied intensively. Interestingly, Cdc42-deficient mice demonstrated the critical function of Cdc42 for normal morphogenesis and functioning of many organ systems including the immune, cardiac, and nervous systems as well as the cartilaginous tissue.

In conclusion, we present an adult female with RP, autoinflammation, and recurrent infections in whom genetic analysis revealed describe a stop-loss mutation in *CDC42*. The phenotype showed similarities to the other reported patients with C-terminal *CDC42* mutations, but unlike those, she did not present at neonatal age, nor did she suffer from severe cytopenias and life-threatening inflammation during childhood. Mutations in the C-terminal region of *CDC42*, therefore, do not necessarily cause severe neonatal-onset inflammation, but can cause later-onset, longstanding systemic autoinflammation and necrotizing vasculitis. This particular clinical phenotype expands the growing spectrum of disease caused by *CDC42* mutations.

## Supplementary Information

Below is the link to the electronic supplementary material.

Supplementary file1 (XLSX 18 KB)

## Data Availability

The datasets generated during and/or analyzed during the current study are available from the corresponding author on reasonable request.
